# Recurrent maternal virilization during pregnancy in patients with PCOS: two clinical cases

**DOI:** 10.1186/s12958-018-0428-9

**Published:** 2018-10-30

**Authors:** M. Deknuydt, A. Dumont, A. Bruyneel, D. Dewailly, S. Catteau-Jonard

**Affiliations:** 1 Service de Gynécologie Endocrinienne et Médecine de la Reproduction, Hôpital Jeanne de Flandre, CHRU Lille, 2 Avenue Eugène Avinée, 59037 Lille, France; 2 Centre Hospitalier de Tourcoing, 155 rue du Président René Coty, 59200 Tourcoing, France; 3 Centre Hospitalier Régional Universitaire de Lille, 2 Avenue Oscar Lambret, 59037 Lille, France

**Keywords:** Polycystic ovary syndrome, Luteinic cyst, Luteoma, Pregnancy, Virilization, Hyperandrogenism, Gestational diabetes, Insulin resistance

## Abstract

**Background:**

Maternal virilization during pregnancy is a rare phenomenon. Polycystic ovary syndrome (PCOS), luteoma and luteinic cysts are the most frequent and benign etiologies. This article presents two cases of recurrent maternal virilization during pregnancy.

**Clinical cases:**

Our reported cases were young women with Afro-Caribbean and Nigerian origins. Data were collected by history-taking, clinical examination, laboratory investigations, transabdominal ultrasonographic examination and Magnetic Resonance Imaging. Both patients were diagnosed with PCOS according to the Rotterdam criteria. During each of their pregnancies they both developed an explosive hirsutism, a deepening in the voice, a clitoromegaly. Gestational diabetes occurred during pregnancies. There was no fetal virilization, despite raising androgen levels, more than tenfold to normal. Improvement of hirsutism and normalization of androgens were described in postpartum.

**Conclusion:**

Only few cases of maternal virilization during pregnancy were reported in literature and even fewer concern recurrent and bilateral ovarian etiology. Hyperplasia of ovarian theca cells seems to be the most likely explanation, which would suggest that PCOS belongs to a spectrum of abnormal reactivity of the ovary to human Chorionic Gonadotrophin (hCG) stimulation along with luteoma and luteinic cyst of pregnancy.  Insulin resistance could worsen hyperandrogenism but is not enough to explain virilization. Treatment should focus on protecting the fetus of possible virilization as well as its mother, but also on preserving the subsequent fertility in both.

## Introduction

Physiologically, pregnancy comes with biological hyperandrogenism [1]. Many mechanisms have been described to explain this hyperandrogenism. First the increase of total testosterone results from the increase of Sex Hormone Binding Globulin (SHGB). The increase of free testosterone serum concentration may be the consequence of the stimulation by human Chorionic Gonadotrophin (hCG), an adrenal influence and a reduced renal clearance of testosterone during pregnancy [2]. Thanks to many protective mechanisms, maternal virilization during pregnancy is uncommon. PCOS is the major cause of hirsutism in women, but is rarely responsible on its own for a maternal virilization during pregnancy [3]. Luteinic cysts and luteoma during pregnancy are the two other benign ovarian etiologies of maternal virilization. Potential other causes are ovarian tumors and adrenal etiologies such as adrenal tumor, 21-hydroxylase deficiency and Cushing syndrome [4, 5]. In a recent review of the literature, only 30% of the patients with luteinic cysts were virilized and recurrences were anecdotal [6, 7] as well as with luteoma [8]. Up to date, only few cases of recurrent maternal virilization during pregnancy have been published [7, 9–14], including one with PCOS [4]. We report two cases of recurrent virilization during successive pregnancies, in women having PCOS and gestational diabetes.

## Clinical cases

### Case 1

A 36-year-old Afro-Caribbean woman with PCOS, according to the Rotterdam criteria [15], presented a recurrent virilization syndrome during four pregnancies. The only known past medical history was a type 2 maternal diabetes. Menarche occurred at 13 years old, with irregular cycles but no sign of hyperandrogenism. Ovulation disorder persisted in adulthood, but the patient had four spontaneous pregnancies. The body mass index was 28 kg/m^2^. All four pregnancies are described in Table 1. Pregnancies were complicated by gestational diabetes. During the first pregnancy, a deepening in the voice and an enlargement of feet were described. Most of the symptoms spontaneously resolved after delivery except the deep voice. During the 3 next pregnancies, hirsutism and signs of virilization started again as described in Table 1. A right adnexal torsion required an adnexectomy in the postpartum of the third pregnancy. Enlargement of the face, hands and feet (two sizes of shoe), deep voice and clitoromegaly persisted after the fourth pregnancy. Only hirsutism decreased over the following weeks in postpartum. Unfortunately, no picture of patient was available. The patient’s history excluded iatrogenic causes, such as anabolic agents. Newborns did not have clitoromegaly nor ambiguous genitalia.

**Table 1:** Anamnesis (case 1)

Pregnancy	Age	Signs of virilization	Complications during pregnancy	Delivery	Postpartum
1st	27	- deep voice - enlargement of feet	- gestational diabetes	- 36 weeks’ gestation (w.g) - girl 2440 g non virilized	- breastfeeding limited to one week because of a delay in lactation
2nd	32	- deep voice - facial hair, hair growth on limbs and abdomen - clitoromegaly - face and hands edema		- 34 + 5 w.g - girl 3215 g non virilized	- breastfeeding limited to few days because of a delay in lactation
3rd	35	- deep voice - clitoromegaly - severe hirsutism	- gestational diabetes treated with insulin - menace of preterm delivery at 25 and 32 w.g (treated with corticosteroids)	- 32 + 2 w.g - boy 2250 g with hyaline membrane disease	- right adnexectomy for adnexal torsion - pathological analysis non-contributive (necrosis)
4th	36	- deep voice - clitoromegaly - explosive hirsutism Ferriman and Gallwey score = 20.	- gestational diabetes treated with insulin - menace of preterm delivery at 28 + 5 w.g (treated with corticosteroids)	- 37 w.g - non virilized girl	- sub torsion of the left adnexal

*w.g* weeks’ gestation, *FaG* Ferriman and Gallwey score

Serum androgen concentrations were measured in the postpartum of the second and third pregnancies and were normal. A hormonal follow up was initiated with the 4th pregnancy, in order to control the androgens’ levels. Blood investigations revealed elevated androgens’ concentrations during the 1st trimester of 4th pregnancy with a peak at the end of the pregnancy (Table 2). A spontaneous decrease in testosterone and ∆4-androstenedione (∆4) levels was observed 2 weeks after the delivery and a complete resolution a month after postpartum. An adrenal etiology was excluded during the second pregnancy, based on normal concentrations of dehydroepiandrosterone sulfate (2.8 μmol/l), 17-hydroxyprogesterone (2.1 ng/ml) and urinary free cortisol (19 μg/l). Acromegaly was also excluded based on normal Insulin-like growth factor-1 (IGF1).

**Table 2:** Evolution of the hormonal profile during and after the 2nd, 3rd and 4th pregnancies (Case 1)

	P2: 1st m. pp	P2: 3rd m. pp	P3: 1st m. pp	P4: 1st m.	P4: 5th m.	P4: 6th m.	P4: 7th m.	P4: 8th m.	P4: 15 days pp	P4: 17 days pp	P4: 1st m. pp	P4: 5th m. pp
Testosterone (0,14–0,58 ng/ml)^a^	0.17	0.12		*0.75*	*2.02*	*3.60*	*10.38*	*19.05*	*13.3*	*6.46*	0.14	0.22
∆4 (0,7–1,8 ng/ml)^a^	0.9		0.26						*43.10*	*15.31*		0.81
SHBG (21,4–161,8 nmol/l)^a^					29.4	18.6	37.6	27.7	25.8	26.2		

*P* pregnancy, *m* month, *pp* postpartum

^a^ *Normal values in women in early follicular phase*

**Fig. 1:**
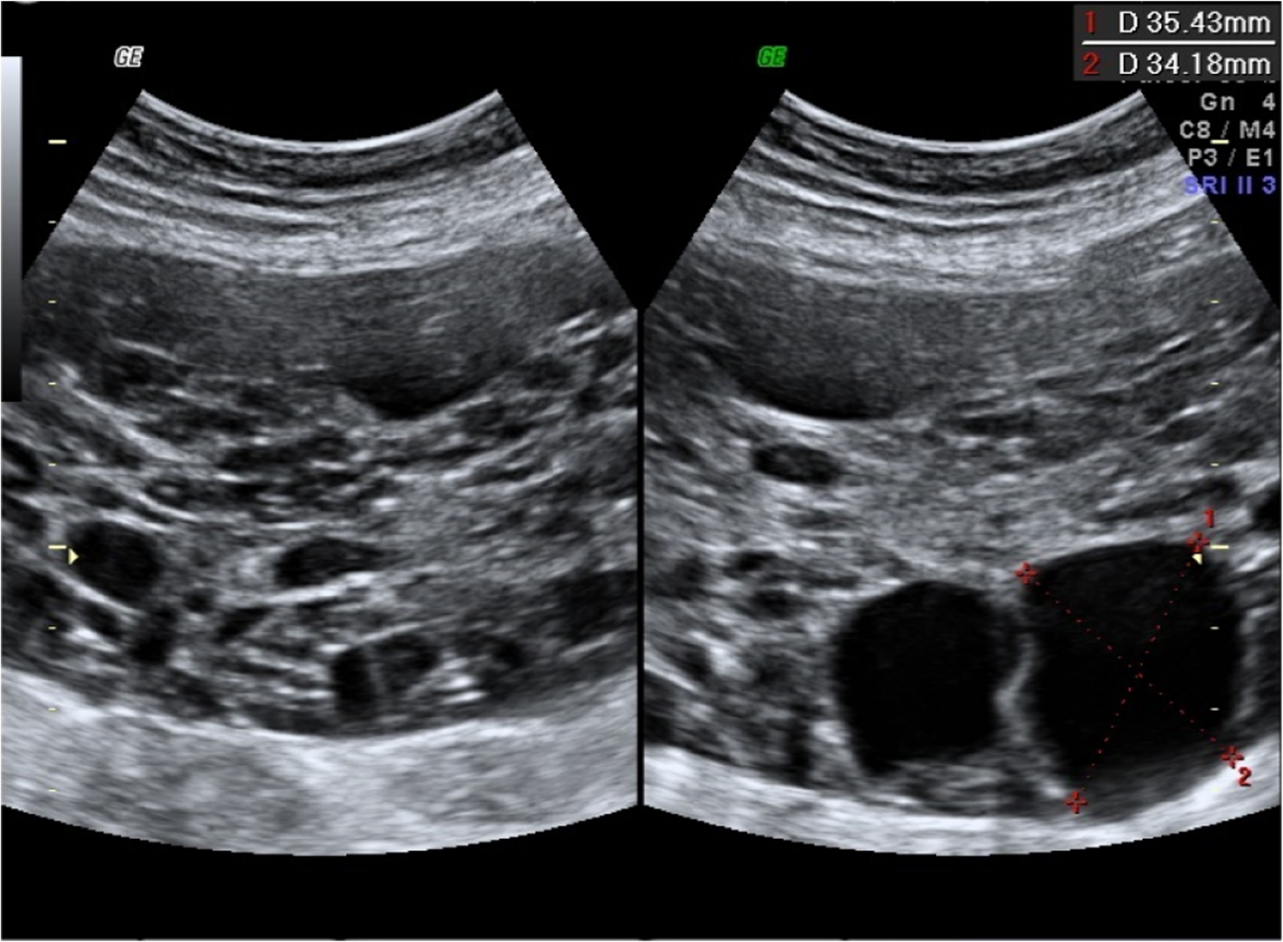
U/S of the left ovary in the immediate postpartum (Case 1)

A pelvic ultrasound (U/S), performed in the first trimester of the 4th pregnancy to exclude ovarian causes of hyperandrogenism (luteoma, luteinic cyst or malignant causes), described the single left ovary with an area of 9,9 cm^2^ and multiple microfollicles. At the same time, a Pelvic Magnetic Resonance Imaging (MRI) confirmed the polycystic ovarian pattern. In the immediate postpartum, another U/S described the polycystic aspect of the left ovary, that was a larger and an anechogenic cyst of 3.5 cm diameter (Fig. 1).

**Fig. 2:**
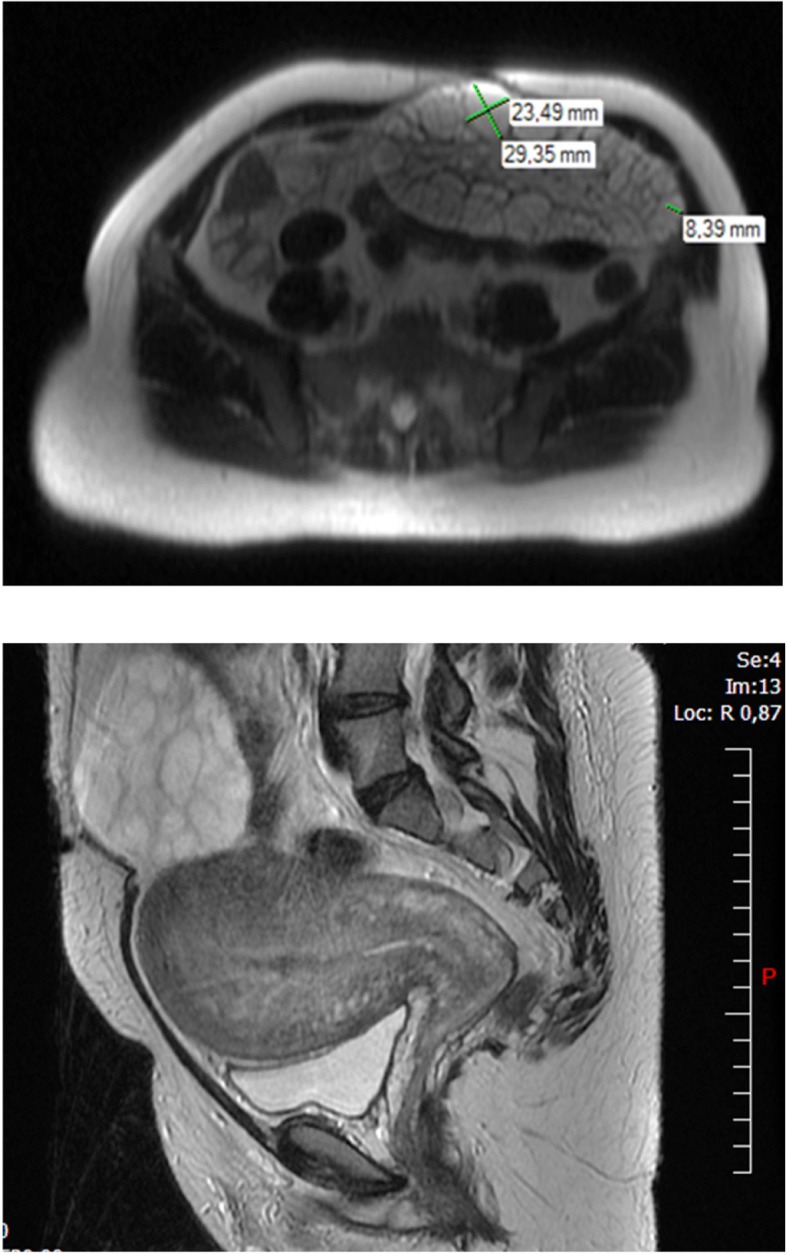
Pelvic MRI performed one month postpartum, left ovary (Case 1)

A second MRI was performed 1 month postpartum because of pelvic pain, suggesting a subtotal ovarian torsion (Fig. 2a and b). The MRI described an enlarged left ovary of 16.5 × 8.2 × 10 cm, a polycystic aspect and an anechogenic cyst up to 3 cm. No detectable solid mass was observed. The ovary was twisted with a lack of vascularization within some parts of parenchyma. The MRI was non-contributive for this area. The clitoromegaly and the deep voice remained but the patient was lost to follow-up.

### Case 2

A 37-year-old woman originating from Niger with no medical history, presented a recurrent virilizing syndrome during her two pregnancies (Table 3). A PCOS was diagnosed before the pregnancies. During the first pregnancy, the patient presented a hirsutism, an enlargement of hands and feet. Blood investigations at the end of the first pregnancy excluded differential diagnoses such as an adrenal etiology based on normal concentrations of dehydroepiandrosterone sulfate, 17-hydroxyprogesterone and urinary free cortisol (320 μg/24 h). A normal level of IGF1 (0.4 ng/l) excluded an acromegaly. After the first pregnancy, the hirsutism decreased. During the second pregnancy, symptoms of virilization worsened as described in Table 3. No picture of patient was available. Gestational diabetes occurred during pregnancies. Newborns did not have clitoromegaly nor ambiguous genitalia.

**Table 3:** Anamnesis (Case 2)

Pregnancy	Age	Signs of virilization	Complications during pregnancy	Delivery	Postpartum
1st	35	- hirsutism - clitoromegaly - enlargement of faces and extremities	- gestational diabetes - Pre-eclampsia	- 34 + 2 w.g - boy 2050 g	- breastfeeding for 6 months
2nd	36	- increase of hirsutism - deep voice - clitoromegaly	- gestational diabetes	- caesarean for caudal presentation - normal weight boy	

A hormonal follow up was designed every 2 months of the second pregnancy, showing an increase in testosterone, ∆4-androstenedione and SHBG concentrations during the 2nd trimester. Testosterone concentration rapidly returned to normal in post-partum (Table 4).

**Table 4:** Biological profile of androgens during the 1st and 2nd pregnancy (Case 2)

	P1: 7th m.	P2: 4th m.	P2: 6th m.	P2: 8th m.	P2: 4th m. pp
Testosterone (0,14–0,58 ng/ml)^a^	*6.15*	*3.9*	*5.79*	*10.78*	0.24
∆4 (0,7–1,8 ng/ml)^a^	*24.8*	*6.6*			
SHBG (21,4–161,8 nmol/l)^a^		82.7	*231*	*315*	14
AMH (11–30,3 pmol/l)^a^					*56,5*

*P* pregnancy, *m* month, *pp* postpartum

^a^Normal values in women in early follicular phase

A pelvic MRI, performed during the 2nd pregnancy, did not find any adrenal abnormality but only polycystic ovaries. In addition, the caesarean showed a macroscopic aspect of “polycystic” ovaries. The hirsutism improved within weeks postnatally and completely disappeared. Only the clitoromegaly and the deep voice remained. The patient was also lost to follow-up.

## Discussion

The originality of these two cases is the recurrence of a virilizing syndrome in successive pregnancies, complicated with a gestational diabetes. The origin remains imprecise although the spontaneous regression suggests a benign etiology. The investigation of the adrenal and somatotrope axes excluded an adrenal cause and acromegaly. An ovarian abnormality was then most likely to be responsible for these cases of virilization.

Luteinic pregnancy cysts can cause maternal virilization. They occur preferentially in young, Caucasian women, mostly primiparous, with multiple or molar pregnancies with high levels of hCG. The literature describes co-morbidities such as PCOS, dysthyroidism or gestational diabetes [6]. Only 30% of patients with luteinic pregnancy cysts have clinical signs of virilization [5]. High androgen concentrations without fetal virilization is usually observed [5, 6]. At U/S, luteinic cysts are often bilateral, anechogenic and their largest diameter varies from 1 to 3 cm [16]. The anatomopathological exam describes luteinization and hypertrophy of the internal layer of the ovarian theca [7]. In our first case, U/S and MRI found an anechogenic cyst of 3 cm, associated with polycystic ovaries. In our second case, only the aspect of polycystic ovaries was described on the MRI. In addition, none of our patients experienced multiple or molar pregnancies. Therefore, the diagnosis of luteinic cysts seems unlikely in our patients.

Luteoma is among the most common benign ovarian tumors that may be associated with maternal hyperandrogenism. It is described on U/S as an ovarian anechogenic mass with net limits, and a thick capsule. At histopathology, large polyhedral and eosinophilic cells with few or no lipids are found [5, 17]. Biological hyperandrogenism is described to be up to seventy-fold higher than normal. In the literature, 25 to 35% of mothers with luteoma are virilized as well as 2/3 of their new-born daughters [5]. Generally, luteoma is characterized by a spontaneous resolution of hyperandrogenism within weeks after delivery, although some symptoms like the deep voice and the clitoromegaly may remain [14]. As the consequence of the inhibitory effect of androgens on the mammary gland, lactation may be delayed [18]. Several data in our first case suggest luteoma as the potential cause of virilization. First, luteoma occurs mostly in Afro-Caribbean women with pre-existing PCOS [5, 13]. The appearance of clinical symptoms, the persistence of a deep voice and clitoromegaly also supports this diagnosis. Unfortunately, neither the imaging nor the anatomopathological analysis of the resected ovary was contributive because of the ovarian necrosis secondary to the adnexal torsion. Nevertheless, luteoma was the most likely diagnosis and our first case would be the 6th reported observation of recurrent luteoma in pregnancy [4, 9, 12–14].

If it were not the case, and in our second patient, an aggravation of the pre-existing PCOS during pregnancy could be suggested. Serum testosterone levels during pregnancy are higher in women with PCOS than in controls [19–21]. However, in the majority of cases, this has no clinical repercussion. However, few cases of virilization in pregnant PCOS patients were described in literature [3–5] and they occurred during the first trimester of pregnancy, while in our patients, the onset of virilization occurred during the 2nd trimester of pregnancy and it was rapid, which is different from the PCOS cases previously reported. The cause of PCOS aggravation remains enigmatic. “LH-like” action of hCG was evoked in the pathophysiology of benign ovarian hyperandrogenism of pregnancy. The cells of the ovarian theca are sensitive to hCG and its “LH-like” effect, which lead to a reactional ovarian hyperplasia [4]. However, the peak of hCG appears in the first trimester of pregnancy whereas the symptoms seem to appear mainly in the 2nd or 3rd trimester of pregnancy.

Other authors discuss the role of insulin and/or IGF1, which together with LH, increase androgen biosynthesis by ovarian theca cells [4, 22]. Accordingly, in our cases, the excess of testosterone was associated with overweight and gestational hyperglycemia. The worsening effect of hyperinsulinism in PCOS is well known [23], due to insulin resistance that increases during pregnancy. However, this occurs in many cases of PCOS without resulting in clinical hyperandrogenism. Interestingly, however, recent studies reported that insulin resistance associated to PCOS could contribute to hyperandrogenism by inhibiting placental aromatase and decreasing conversion of androgens to estrogens [24, 25]. Such a phenomenon could be amplified in some patients, for unknown reason(s). It has been hypothesized that pregnancy hCG, that persistently overstimulates PCOS ovaries in pregnancy, can be associated with CYP19A1 (aromatase) deficiency and placental inability for androgen conversion [26]. Alternatively, in PCOS, the Anti-Müllerian Hormone (AMH) is high [27] and it is known to inhibit aromatase activity [28]. Even if AMH tends to decrease during pregnancy, its serum level remains higher than in controls [29]. We can therefore suspect an inhibition of placental aromatase by excessive production of AMH in our cases. Unfortunately, in our patients, we have no data about serum AHM during pregnancy and placental aromatase could not be measured.

Finally, there could be the same mechanisms that are still not understood as those that occur in ovarian hyperstimulation in patients with PCOS, involving growth factors such as vascular endothelium Growth Factor [30].

## Conclusion

Even though PCOS is frequent, virilization during pregnancy is rare. The physiopathology remains to be elucidated. Hyperplasia of ovarian theca cells seems to be the most likely explanation, which would suggest that PCOS belongs to a spectrum of abnormal reactivity of the ovary to hCG stimulation along with luteoma and luteinic cyst of pregnancy [4]. Insulin resistance and low rate of SHBG could worsen hyperandrogenism but is not enough to explain virilization. Finally, a placental dysregulation with partial aromatase deficiency can be suggested. The question of how avoiding recurrence in a future pregnancy remains open. A biological follow-up and U/S maternal monitoring could help preventing complications and avoid deleterious adnexectomy. As far as the child prognosis is concerned, based on literature data [31] and the fact that hyperandrogenism occurred rather lately, the risk of virilization of a female fetus is likely very low. Conversely, caution should be paid to the risk of developing PCOS and metabolic syndrome at adolescence. Recently, Filippou et al. [32] suggested that elevated maternal testosterone levels during pregnancy could predict an ovarian dysfunction in adult daughter of PCOS women. Therefore, treatment should focus on protecting the fetus from possible virilization as well as its mother, but also on preserving the subsequent fertility in both.

## Abbreviations

∆4: Androstenedione
AMH: Anti-Müllerian Hormone
FaG: Ferriman and Gallwey score
hCG: Human Chorionic Gonadotrophin
IGF1: Insulin-like Growth Factor-1
LH: Luteinizing Hormone
m: Month
MRI: Magnetic Resonance Imaging
P: Pregnancy
PCOS: Polycystic Ovary Syndrome
pp: Postpartum
SHBG: Sex Hormone Binding Globulin
U/S: Pelvic Ultrasound
w.g: Weeks’ gestation
